# Inhibition of γ-secretase in adipocytes leads to altered IL-6 secretion and adipose inflammation

**DOI:** 10.1080/21623945.2020.1788235

**Published:** 2020-06-30

**Authors:** David P. Sparling, Nile McCullough, Utpal Pajvani, Mary Beth Humphrey

**Affiliations:** a Department of Pediatrics, University of Oklahoma Health Sciences Center, Oklahoma City, OK, USA; b Department of Medicine, Columbia University, New York, NY, USA; c Department of Medicine, University of Oklahoma Health Sciences Center, Oklahoma City, OK, USA

**Keywords:** Adipocyte, gamma-secretase, IL-6, macrophage, inflammation

## Abstract

Adipocyte-mediated inflammatory signalling has been proposed to alter adipose physiology in obesity and Type 2 diabetes mellitus. Novel targets for alteration of inflammatory signalling are needed to improve obesity-related outcomes. The γ-secretase enzyme complex has been suggested to play a role both in adipocyte function as well as in immune regulation. We hypothesized that adipocyte-specific γ-secretase inhibition could alter the inflammatory makeup of adipose tissue. We found that genetic blockade of γ-secretase in adipocytes leads to a decrease in *EMR1* (F4/80) expression, as a marker of macrophage presence, in adipose tissue without changes in expression of markers of other inflammatory cell types. To explore the mechanism by which adipocytes can alter macrophage function *in vitro*, fully differentiated 3T3-L1 adipocytes were treated with a γ-secretase inhibitor in the presence of lipopolysaccharide (LPS) and transcription of *IL6* and *ccl2* (MCP1) were quantified. IL-6 expression and secretion were significantly inhibited by γ-secretase blockade, with little effect on MCP1. Preconditioned media from 3T3-L1 adipocytes treated with a γ-secretase inhibitor also alters macrophage activation but did not affect macrophage translocation *in vitro*. Therefore, γ-secretase inhibition in fully differentiated adipocytes can alter IL-6 signalling to macrophages, consistent with our hypothesis that that γ-secretase is involved in adipocyte-initiated inflammatory signalling cascades.

## Introduction

The proximal cause of obesity-induced insulin resistance is excessive adipose mass [1] and one factor that can affect adipose function is inflammation. As there is a broad spread in pathophysiology, inflammatory signalling, and response to treatment in obesity, new signalling cascades and drug targets are needed to prevent the long-term morbidities that can arise in obese youth and persist into adulthood [2–6]. The adipocyte itself has been implicated in direct regulation of adipose inflammation [7–9]. Therefore, novel pathways regulating adipocyte-initiated inflammation could provide new avenues of treatment for obesity and type 2 diabetes (T2DM).

The γ-secretase enzyme complex is a heterotetramer of four obligate components – Nicastrin, PSEN1, APH-1, and PEN-2 – that regulates intramembranous cleavage of type-I transmembrane proteins termed regulated intramembrane proteolysis. This process releases the C-terminal intracellular domain of the transmembrane protein, which may go on to mediate downstream effects [10]. Several immune signalling cascades utilize γ-secretase and regulated intramembrane proteolysis, including Notch, CD44, and TREM2 [11–14]. Notch signalling, associated with cell-fate decisions [14] and whole-body glucose metabolism [15], is a classic example of both γ-secretase function and the consequences of its disruption [16]. Upon activation, Notch undergoes regulated intramembrane proteolysis by γ-secretase, releasing the Notch intracellular domain (NICD). NICD translocates to the nucleus and activates Rbp-Jκ-dependent transcription of the *Hes* and *Hey* transcription factor families, which regulate the variety of cellular responses to Notch activation.

The roles of γ-secretase broadly and Notch specifically in adipose function are unclear. For example, Notch activation induces adipogenesis, but γ-secretase inhibition (GSI) leads to the same effect [17,18], suggesting either time-dependent and/or Notch-independent signalling events are required. Previous data directly implicated γ-secretase in the regulation of insulin signalling in adipose tissue, but in a Notch-independent manner [19]. Adipocyte-specific blockade of γ-secretase activity was achieved by flox-mediated knockout of Nicastrin (A-Nicastrin) via cre-recombinase under the control of the adiponectin promoter. A-Nicastrin mice had no change in whole-body insulin sensitivity compared to cre-negative littermate controls, under both chow- and high-fat-fed conditions. However, non-esterified free fatty acids were increased following Nicastrin knockdown, consistent with decreased adipose insulin sensitivity. Other work has suggested inhibition of γ-secretase can lead to decreased obesity and increased browning of white adipose tissue [20]. These data show that γ-secretase is active in adipocytes and may have diverse roles in both the development and functioning of mature adipocytes, in both Notch-dependent and Notch-independent cascades.

A novel function of γ-secretase could be in regulating immune signalling cascades initiated by adipocytes themselves in either Notch dependent or independent manners. For example, the Notch cascade is involved in the development and regulation of multiple immune cell types [21]. Alternatively, Nicastrin knockdown directly decreases dendritic cell antigen presentation [22]. Several inflammatory cytokines have been shown to be secreted by adipocytes, and have been tied to obesity or alterations in adipocyte function [9,23–25]. Among these, MCP1 and IL-6 have been well characterized in a variety of cell types. Both MCP1 and IL-6 signalling are regulated, at least in part, at the transcriptional level, but cytokines such as IL-6 can be regulated by their secretory machinery as well [26]. Obesity is thought to lead to increased Toll-like receptor 4 (TLR4) stimulation in adipocytes via a variety of ligands [27], and expression of both IL-6 and MCP1 by adipocytes can be induced by inflammation [28]. Therefore, determining both the cell and cascade-specific effects and mechanisms of γ-secretase inhibition are needed to determine what role γ-secretase blockade might have in possible therapies.

We hypothesized that γ-secretase is integral to adipocyte-initiated inflammatory signalling cascades. We first analysed stored whole adipose from the previously characterized cohort of animals that had adipocyte-specific blockade of γ-secretase activity, with concomitant altered adipocyte insulin sensitivity [19]. Samples from the cohort were re-examined for alterations in inflammatory gene expression. However, analysis of our initial studies was complicated by the complex cellular makeup, including not only adipocytes, but the resident lymphocytes and other components of the stromal vascular fraction (SVF), of whole adipose tissue. Therefore, while γ-secretase has been implicated in immune signalling cascades and adipocytes separately, its specific role in adipocyte-initiated inflammatory signalling was further studied *in vitro* in 3T3-L1 adipocytes, limiting the noise from gene transcription and translation from the SVF. We determined whether γ-secretase inhibition is able to decrease resident adipose tissue macrophages and/or alter their activation. We further studied adipocyte-specific alterations and mechanisms of known adipokines’ secretion, exploring an initial pathway in which γ-secretase functions in regulation of adipose tissue inflammation.

## Materials and methods

### Experimental animals

Results described were obtained from stored samples from a previously described cohort [19], wherein all procedures had been approved by the Columbia University Institutional Animal Care and Utilization Committee. In short, adiponectin-cre [29] animals were crossed with *Nicastrin*^flox/flox^ [15] mice, all on the *C57/BL6* background, to generate adiponectin(cre); *Nicastrin*^flox/flox^ (*A-Nicastrin)* animals. Animals were weaned to either standard chow (Purina Mills 5053) or high-fat diet (18.4% calories/carbohydrates, 21.3% calories/protein and 60.3% calories/fat derived from lard; Harlan Laboratories, TD.06414).

### Cell culture and adipocyte transfection

3T3-L1 preadipocytes (ATCC) were maintained in DMEM, high glucose GlutaMAX-I media (Gibco) with 10% Calf serum (Gibco) and differentiated according to normal specifications. RAW 264.7 macrophages (ATCC) were maintained under normal tissue culture procedures. Where specified, cells were treated with 200 nM *N*-[(1 *S*)-2-[[(7 *S*)-6,7-Dihydro-5-methyl-6-oxo-5 *H*-dibenz[*b,d*]azepin-7-yl]amino]-1-methyl-2-oxoethyl]-3,5-difluorobenzeneacetamid (DBZ, Tocris), cycloheximide (Sigma), and/or lipopolysaccharide (LPS, Sigma) added to concentrations noted.

### Quantitative real time-PCR (qRT-PCR)

RNA was isolated from whole adipose tissue via the RNeasy Lipid mini-kit (Qiagen); RNA from whole cell lysates was obtained with the RNAeasy mini-kit (Qiagen). cDNA was generated from mouse adipose RNA extract as described [19]; cDNA from 3T3L1 adipocyte RNA extracts was generated using the High Capacity cDNA Reverse Transcription Kit (Applied Biosystems). Quantitative real-time PCR was performed on a CFK96 Real-Time PCR detection system (Bio-Rad) with the Power SYBR Green PCR Master Mix (Applied Biosystems). Primers for all genes are available on request. Gene transcription was quantified using the ΔΔCt method using TATA Binding Protein (TBP) as a control to determine relative gene expression. Rapid analysis of inflammatory cascade gene expression was performed using a PrimePCR Custom 96 well plate (Bio-Rad) according to manufacturer’s specifications. Genes screened for included *Adam17, Il4ra, Mrc1, Fos, Il1b, IL12a, Il6, Il10, Hes1, Jak1, Jun, Map2k1, Mapk1, Mapk3, Nfkb1, Nfkb2, Nr3c1, Ptpn11, Tnf, Ccl2, Ccl3, Nos2, Socs3, Stat3, Tbp, Gapdh*, and *Hprt*.

### ELISA and immunohistochemistry (IHC)

Cells were lysed in Whole Cell Lysis Buffer (20 mM Tris, pH 7.4 150 mM NaCl, 10% glycerol, 2% Nonidet P-40, 1 mM EDTA, pH 8.0, 0.1% SDS, 0.5% sodium deoxycholate) supplemented immediately prior to use with HALT Protease & Phosphatase Inhibitor Single-Use Cocktail (Roche). ELISAs for mouse MCP1 and IL-6, both secreted into media or from WCL as above, were performed according to manufacturer’s specifications (R&D Systems). For IHC whole adipose tissue was fixed in Z-fix (Anatech), paraffin embedded, sectioned and stained with H&E and F4/80 through the Advanced Tissue Pathology and Imaging Core in the Diabetes Research Centre at Columbia University Irving Medical Centre.

### Macrophage migration

Media from 3T3L1 adipocytes pre-treated with ± DBZ overnight and then ± LPS for 6 hours was collected, filter sterilized, and utilized in a QCM 24-Well Colorimetric Cell Migration Assay (Millipore) with RAW 264.7 macrophages; at the end of the assay the insert was secondarily stained with DAPI and cells were manually counted.

### Statistical analysis

All results are reported as mean ± SEM. Gene expression levels were compared using Students t-test. Experiments utilizing CHX/LPS or DBZ/LPS treatments were analysed by 2-way ANOVA with post-hoc Tukey HSD test. P values of <0.05 were considered significant.

## Results

### Decreased γ-secretase activity alters adipose tissue macrophages

**Figure 1.**
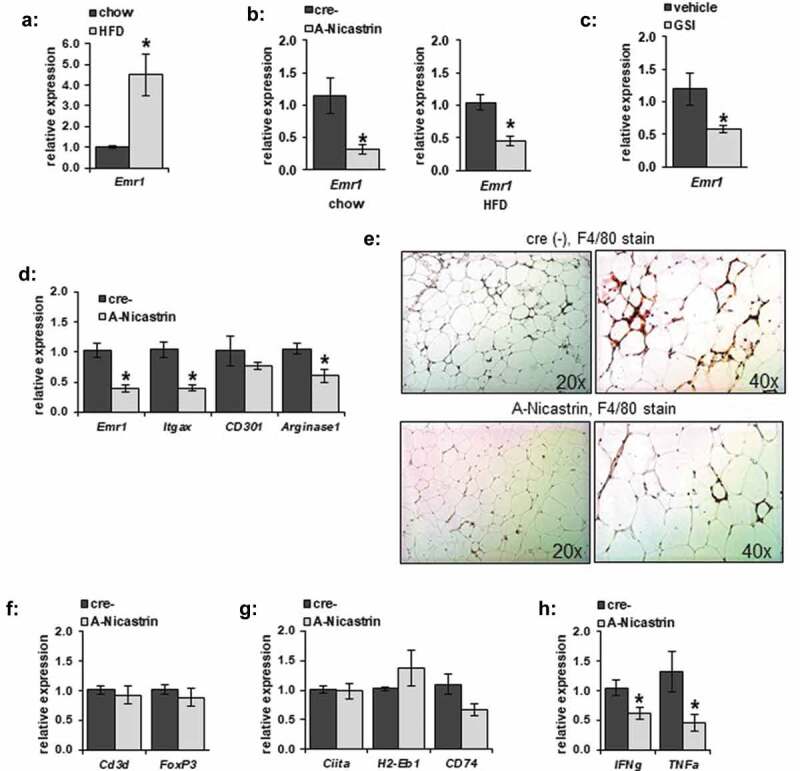
Adipocyte-specific γ-secretase blockade alters adipose inflammation. (a) Relative gene transcription of F4/80 (*Emr1*) was examined from eWAT of C67/Bl6 mice maintained on chow or HFD. (b) F4/80 transcription from eWAT from A-Nicastrin mice and their cre (-) littermate controls maintained on chow or HFD. (c) F4/80 expression was measured from eWAT adipose depots from wild type chow-fed animals treated with vehicle or 2 mg/kg GSI for 5 days (d) Relative transcription of markers of macrophage activation were compared between HFD-fed A-Nicastrin mice and their cre (-) littermate controls. (e) Representative images from adipose stained for macrophages via anti-F4/80 IHC in HFD-fed A-Nicastrin mice and their cre (-) littermate controls (f) Relative expression of total T-cell and T-reg cell markers, (g) MHC-II associated proteins, and (h) inflammatory cytokines from HFD-fed A-Nicastrin animals and their cre (-) littermates are shown (n = 7/condition, error bars are ± SEM, * = p < 0.05)

We previously found that adipocyte-specific knockout of γ-secretase activity, by ablation of the non-redundant Nicastrin subunit (A-Nicastrin mice) decreased adipose insulin sensitivity [19]. However, those initial studies did not examine the local changes to the SVF within the adipose tissue. We, therefore, examined several markers of the SVF from the epididymal fat pad (eWAT). *Emr1* (F4/80), a well-known marker of adipose tissue macrophages, which increases in response to HFD (Figure 1(a)), was lower in A-Nicastrin eWAT adipose tissue compared to that from cre-negative littermates independent of diet (Figure 1(b)). GSI was able to similarly inhibit *EMR1* in WT animals (Figure 1(c)). Thus, inhibition of γ-secretase chemically or by disruption of the complex reduces macrophage markers in adipose tissue.

We next determined what immunological cell lines might be disrupted at the transcriptional level in adipose tissue from A-Nicastrin mice. In eWAT from HFD-fed animals, transcription of markers of M1 and M2a polarization, *Itgax* (Cd11 c) and *Arginase1* respectively, were decreased in a similar fashion to F4/80 (Figure 1(c)); CD301, another M2 marker, was not significantly affected. Histologically, F4/80(+) cells and crown-like structures in HFD-fed A-Nicastrin adipose were reduced compared to WT controls (Figure 1(d)). These observed macrophage changes were not accompanied by a decrease in expression of a broad marker of T cells (*Cd3d*) or T regulatory cells (*FoxP3*) (Figure 1(f)), as noted by expression of *Cd3d* and *FoxP3* respectively. Transcription of MHCII markers *Ciita* as well as *H2-Eb1* and *CD74* were unaffected in A-Nicastrin mice (Figure 1(g)). *IFNg* and *TNFa* transcription were decreased in A-Nicastrin mice, likely reflective of a decrease in local pro-inflammatory cytokine production.

### γ-secretase blockade alters basal adipocyte inflammatory cytokine expression

**Figure 2.**
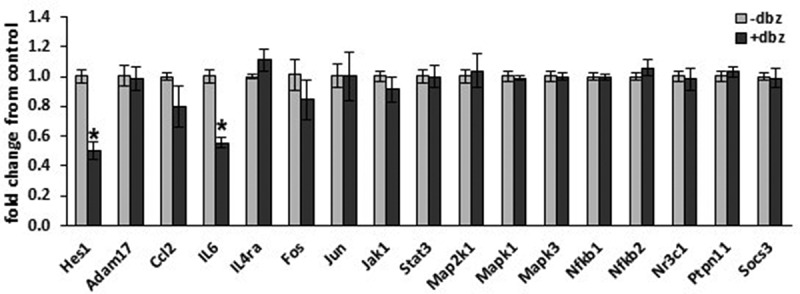
Adipocyte inflammatory gene expression is altered in response to γ-secretase blockade. Fully differentiated 3T3-L1 adipocytes were incubated in the absence or presence of γ-secretase-inhibitor DBZ (200 nM) for 18 hours. Total RNA was purified and gene expression analysed by the ΔΔCt method on a PrimePCR Custom 96 well plate (n = 5–6/condition, error bars are ± SEM, * = p < 0.05)

Due to the complex makeup of adipose tissue, we specifically focused on the adipocyte to determine changes in inflammatory gene expression caused by GSI *in vitro*. 3T3-L1 adipocytes were incubated overnight in the absence or presence of 200 nM DBZ, a potent γ-secretase inhibitor. Total RNA was purified and analysed for a broad set of genes involved in inflammatory signalling by qRT-PCR (Figure 2). Consistent with inhibition of Notch signalling known to occur with GSI, *Hes1* expression was significantly down-regulated. Basal *IL6* transcription was significantly decreased by γ-secretase inhibition, whereas basal levels of *ccl2* and several other genes within inflammatory signalling cascades were unaffected. These data indicate that γ-secretase inhibition does not broadly change basal adipocyte inflammatory cytokines or transcription factors but can specifically suppress basal *IL6* transcription.

### γ-secretase blockade can inhibit LPS-induced IL-6 and MCP1 transcription and alters basal and stimulated IL-6 secretion

**Figure 3.**
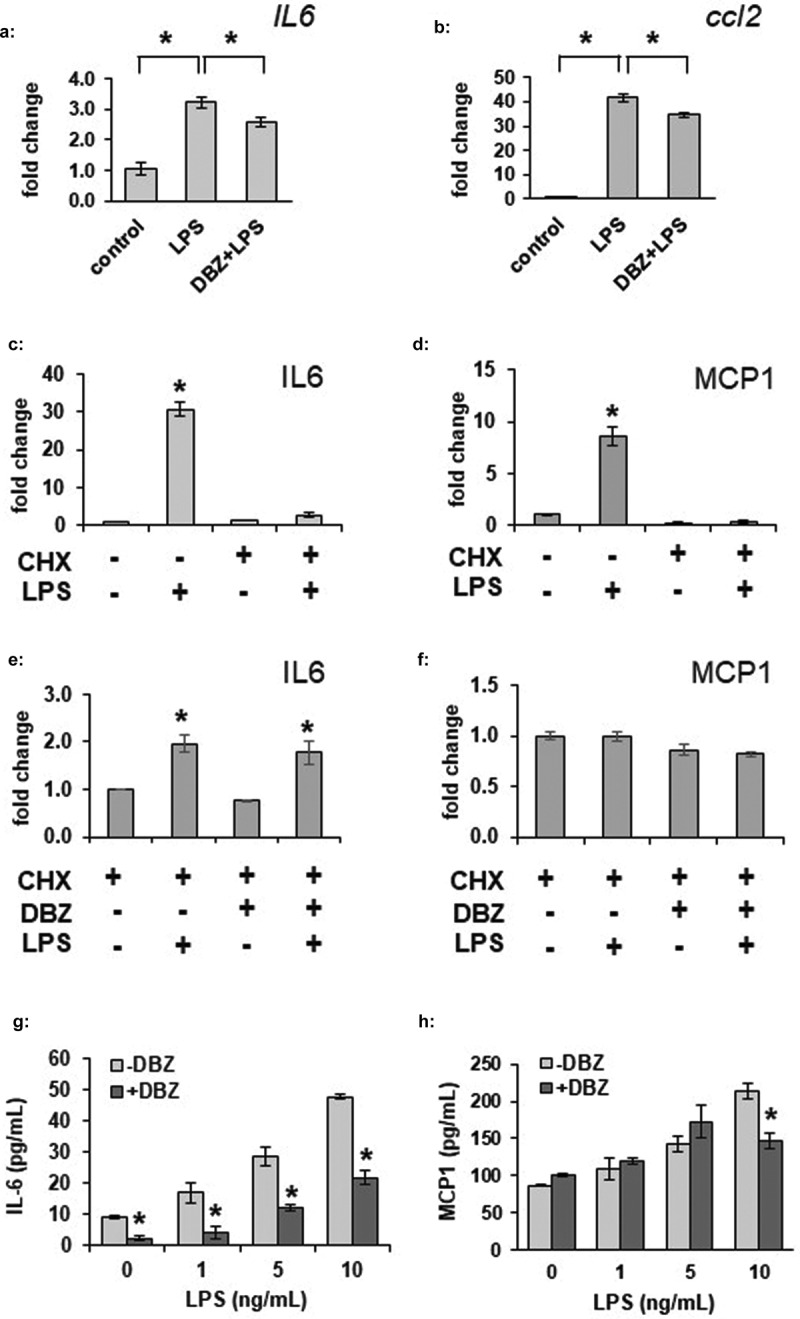
LPS-stimulated adipocyte IL-6 and MCP1 secretion is decreased by γ-secretase inhibition. (a) Fully differentiated 3T3-L1 adipocytes were exposed to LPS at 100 ng/mL for 6 hours in the absence or presence of overnight exposure to 200 nM DBZ. Total RNA was purified from the adipocytes and gene expression for IL-6 (a) or MCP1 (*ccl2*, b) was analysed by the ΔΔCt method, comparing to unstimulated adipocytes (n = 6/condition, error bars are ± SEM, * = p < 0.05). IL-6 (c) or MCP1 (d) secretion was measured by ELISA from conditioned media from LPS-treated 3T3-L1 adipocytes in the absence or presence of a 2 hour pre-incubation with cycloheximide (CHX) (n = 6/condition, fold change from untreated samples, error bars are ± SEM, * = p < 0.05 from untreated media). Media was similarly analysed for IL-6 (e) or MCP1 (f) secretion from LPS-stimulated 3T3-L1 adipocytes treated with CHX that had been previously treated overnight in the absence or presence of 200 nM DBZ. Increasing amounts of LPS were used to stimulate 3T3-L1 adipocytes following the absence or presence of DBZ (200 nM, overnight exposure) and IL-6 (g) or MCP1 (h) levels were assess by ELISA (n = 6/condition, error bars are ± SEM, * = p < 0.05 from the untreated samples.)

LPS is known to induce a variety of inflammatory signals within adipocytes. As basal transcription levels of the cytokine IL-6 were affected by GSI, we sought to determine whether γ-secretase could alter LPS-stimulated transcription of IL-6 or MCP1. We incubated 3T3-L1 adipocytes in the absence or presence of DBZ overnight, followed by stimulation with 100 ng/mL. *IL6* and *MCP1* transcription were significantly increased by LPS stimulation, and partially blocked by GSI (Figure 3(a,b)). To determine if this had an effect beyond the transcriptional level, cytokine secretion was next examined. Stimulated secretion of cytokines such as IL-6 may be regulated at the transcriptional level as well as at via secretory machinery [30]. As expected, cycloheximide pre-treatment inhibited LPS-induced secretion of both IL-6 and MCP1 from adipocytes (Figure 3(c,d)). We wondered if the full effect of GSI was due purely to blockade of transcription and translation of IL6 or MCP1, or if there was also a further blocking effect on stored cytokine secretion through changes in secretory machinery. Pre-treatment with the γ-secretase inhibitor DBZ did not alter the cycloheximide effect even though a small amount of IL-6, but not MCP1, was still secreted (Figures 3(e,f)). A small pool of IL-6 can, therefore, be secreted even without active gene translation, and this pool appears resistant to the effects of GSI. Taken together, these data indicate that γ-secretase inhibition prevents full induction of IL-6 and MCP1 gene transcription, and that secretion of IL-6 and MCP1 are dependent on gene translation in adipocytes.

To determine whether LPS-induced IL-6 secretion could be affected by γ-secretase inhibition, we incubated adipocytes overnight in the absence or presence of DBZ, then stimulated them with increasing amounts of LPS. Pre-treatment of adipocytes with DBZ significantly attenuated adipocyte IL-6 secretion both in the basal and LPS-stimulated states (Figure 3(g)). In stark contrast to the limited effects on adipocyte IL-6 secretion, and despite the significant change in transcription, LPS-induced MCP1 secretion was not drastically affected by GSI (Figure 3(h)), suggesting stored MCP1 might not be dependent on rapid changes in expression in adipocytes.

### LPS-stimulation of IL-6 intracellular expression can persistently be inhibited by GSI

**Figure 4.**
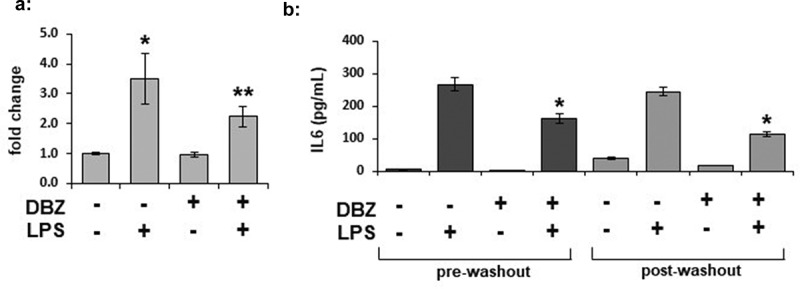
IL-6 expression is regulated by γ-secretase in adipocytes, and secretion can be persistently inhibited by γ-secretase inhibition. Intracellular expression of IL-6 was measured by ELISA from whole cell lysates from 3T3L1 adipocytes pretreated with 200 nM DBZ overnight and then treated with LPS for 6 hours. (n = 6/condition, error bars are ± SEM, * = p < 0.05 from the untreated sample). (b) The persistence of alteration in IL-6 secretion was determined in 3T3-L1 adipocytes treated with DBZ and LPS as previously examined (pre-washout), with the cells then washed 3x with PBS and placed in adipocyte maintenance medium for an additional 18 hours (post-washout); media collected at the 2 time points were analysed by ELISA for IL-6 (n = 6/condition, error bars are ± SEM, * = p < 0.05 from the untreated samples.)

To determine if γ-secretase blockade decreased IL-6 intracellular protein levels in both the basal and LPS-stimulated state, we measured IL-6 levels from 3T3L1 whole cell lysates by ELISA (Figure 4(a)). The LPS-stimulated increase in IL-6 accumulation within the cell was blocked by pre-treatment with DBZ. To determine whether or not the effects of γ-secretase blockade on IL-6 secretion can persist after removal of the LPS stimulus, we incubated adipocytes in the absence or presence of DBZ overnight, then stimulated for 6 hours with LPS. We then washed the cells extensively and incubated them for an additional 18 hours in regular adipocyte maintenance media. LPS levels were decreased following the wash (data not shown). Despite the washout of the LPS and DBZ, the decrease in IL-6 secretion was maintained (Figure 4(b)).

### *γ-secretase blockade does not alter L1-induced macrophage migration* in vitro, *but alters IL-6-induced activation*

**Figure 5.**
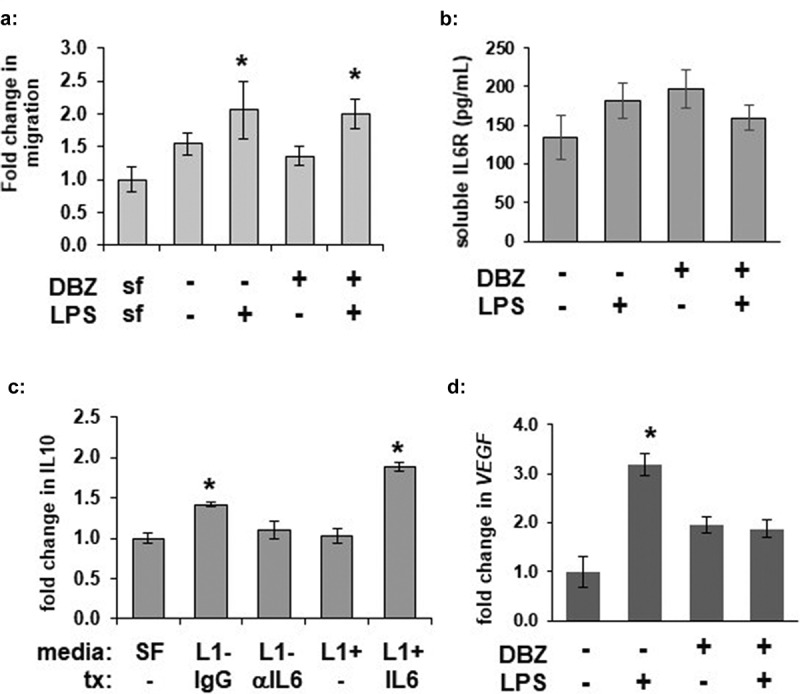
γ-secretase inhibition does not significantly alter adipocyte LPS-stimulated macrophage attraction or soluble IL6 receptor concentration but does alter adipocyte-mediated IL-6 activation of macrophages. (a) RAW 264.7 macrophage translocation was determined in a Boyden chamber after 6 hours of exposure to serum-free (SF) or media from 3T3L1 adipocytes pre-treated with DBZ and/or LPS (n = 6 per condition, * = p < 0.05 from SF control media). (b) Secretion of soluble IL-6 R was measured by ELISA from media from 3T3L1 adipocytes pretreated with 200 nM DBZ overnight and then treated with LPS for 6 hours (n = 6 per condition) (c) Macrophage IL-10 secretion was measured from RAW 264.7 treated with preconditioned media from 3T3-L1 adipocytes pre-treated in the absence (L1-) or presence (L1+) of 200 nM DBZ. Preconditioned media also received further pre-treatment (tx): L1- samples were also incubated with or without IL-6 blocking antibody andL1+ samples were incubated with and without exogenous IL-6. (* = p < 0.05 from – DBZ IgG control or +DBZ samples, respectively.) (d) Fold change of *VEGFa* transcription from RAW 264.7 macrophages following 6 hours of exposure to media from 3T3-L1 adipocytes pre-treated with DBZ or LPS (* = p < 0.05 from control sample, ** = p < 0.05 from +LPS sample)

To determine whether GSI-treated adipocytes could alter macrophage migration, we pre-treated adipocytes overnight in serum-free media with DBZ followed by LPS stimulation to produce conditioned media. The conditioned media was then used media to analysed for its ability to induce macrophage translocation. Media collected from adipocytes following LPS stimulation induced macrophage translocation across a Boyden chamber (Figure 5(a)). Conditioned media from GSI-treated adipocytes stimulated macrophage migration in a similar manner.

IL-6 has previously been suggested to mediate adipocyte inflammatory signalling, and macrophages can secrete IL10 in response to IL-6 [31]. Furthermore, this could be a result of adipose IL-6 *trans*-signalling, wherein soluble IL-6-receptor (sIL6 R) can bind local IL-6 and activate inflammatory signalling in nearby cells expressing gp130 [32]. Soluble IL6 R secretion was not affected by GSI (Figure 5(b)). As IL-6 secretion even in the basal state appeared to be decreased by γ-secretase inhibition, and to avoid complications from residual LPS affecting macrophage function, media from unstimulated adipocytes was used to activate macrophages *in vitro*. Conditioned media from cells pre-treated with a GSI induced significantly less macrophage IL10 secretion, which could be rescued with addition of exogenous IL-6 (Figure 5(c)). Similarly, GSI reduced another IL-6 target, *VEGFa* transcription in alternatively polarized M2d macrophages [33], (Figure 5(d)). These data are consistent with a requirement for γ-secretase function in adipocyte-secreted IL-6 activation of macrophages, likely primarily through alterations of IL-6 expression and secretion.

## Discussion

Adipose inflammation has long been associated with insulin resistance and Type 2 diabetes [34]. Our data further support the hypothesis that signalling cascades within adipocytes participate in regulation of the local inflammatory state. Previous work has shown that γ-secretase blockade in adipocytes leads to decreased insulin sensitivity, in a Notch-independent manner [19]. Our current work suggests that adipocyte γ-secretase blockade simultaneously reduces adipocyte-mediated inflammation, likely by interfering with IL-6 transcription, expression, and secretion, to alter macrophage activation.

When subjecting samples from a previous study to further scrutiny, we found that either genetic or pharmacologic GSI led to a decrease in macrophage markers in adipose tissue. While pharmacologic treatment could lead to non-specific effects of GSI on macrophages, the genetic blockade was specific to adipocytes. Classic markers of macrophage polarization were affected as well, though several other cell lines were not affected when examined at the transcriptional level. Taken together, these data indicated that adipocyte-specific GSI could alter the local inflammatory state of adipose tissue *in vivo*.

IL-6 has been identified as a significant mediator of adipose inflammation [35]. Macrophage IL-6 expression is known to be down-regulated by γ-secretase inhibition [36], and we have observed a similar effect both at the transcriptional and translational levels in adipocytes. This suggests that paracrine inflammatory signalling can be initiated purely by the adipocyte, via a similar mechanism to macrophage regulation, leading to broader adipose inflammation alterations as seen in the A-Nicastrin mouse. The transcriptional regulation likely accounts for at least a part of its persistent effect, as downstream IL-6 function can be regulated through expression of both the cytokine and its endosome machinery. These disparate effects, on both IL-6 expression itself, as well as the secretory machinery, might explain why short-term blockade of γ-secretase leads to a decrease in basal secretion of IL-6, but does not alter its intracellular concentration. Finally, as expected, adipocyte IL-6 can affect macrophage activation, with altered IL-10 secretion and *VEGFa* expression from macrophages exposed to preconditioned media from 3T3L1 adipocytes. These changes were inhibited by prior γ-secretase blockade of the adipocytes, and could be rescued with exogenous IL-6. Taken together, these data suggest that adipocyte γ-secretase blockade can decrease IL-6-mediated activation of macrophages. This correlates with the suggested role of IL-6 in limiting obesity-related insulin resistance in mice [31]. Local alterations in IL-6 paracrine signalling originating in adipocytes might then explain at least a portion of the localized insulin resistance of the A-Nicastrin mice. Most intriguingly, our data is consistent with work on adipose IL-6 *trans*-signalling, wherein soluble IL-6-receptor (sIL6 R) can bind IL-6 and activate inflammatory signalling in nearby cells expressing gp130 [32]. Blockade of IL-6 *trans*-signalling in adipose decreased macrophage recruitment, but did not alter insulin resistance, similar to our *in vitro* findings. While sIL6 R secretion was not altered in this specific adipocyte cell line, decreased downstream signalling through gp130 could occur via the decrease in IL-6 itself. This would correlate with our observed *in vivo* decrease in macrophage recruitment. It is possible that this might occur via altered signalling within CD4 + T-cells, which aid in regulation of obesity-induced inflammation [37], and which can express gp130. Further work into alterations of γ-secretase signalling *in vivo* will be needed to determine if local γ-secretase changes in IL-6 signalling via gp130 are affected in both Notch-dependent (i.e. transcriptional regulation) and Notch-independent (i.e. IL-6-*trans* signalling) manners.

Our data are broadly consistent with the theory of a ‘healthy’ adipose inflammatory state [38,39]. Adipose tissue macrophages (ATMs) have been shown to play different roles in adipose regulation, including activation of pathways increasing lysosomal function, thereby buffering increased local lipid concentrations [40]. Worsening insulin sensitivity and resulting lipolysis are directly associated with alterations in immune response through a variety of pathways [41,42]. We hypothesize that with the worsening insulin sensitivity in adipose tissue following GSI (i.e. with the previously observed increased in NEFA [19],) and these newly observed changes in IL-6 secretion, that GSI can lead to a significant degree of localized adipose insulin resistance.

Our study has some inconsistencies. We initially observed that adipocyte-specific γ-secretase deficiency leads to altered adipose inflammation, implying that adipocytes may regulate both macrophage infiltration and/or activity. However, due to the complexity of the whole tissue, interpretation of the transcriptional studies was difficult. For example, adipocytes are already known to regulate macrophage function via a variety of inflammatory cascades beyond IL-6 [32]. Adipocytes may present antigens via MHC-II molecules, which can also activate CD4 + T-cells and in turn promote ATM M1 polarization and inflammatory cytokine secretion in HFD-induced obesity [43]. We did not observe a change in whole-tissue expression of MHCII-associated genes, despite the observed decrease in ATMs. This might be explained by the possibility of up-regulated MHCII expression on other cells within the adipose tissue SVF. Another possible contradiction involves MCP1. MCP1 has been well characterized for its role in macrophage recruitment and adipose inflammation. However, in our system, MCP1 was only mildly affected in its LPS-stimulated transcription and secretion. Interestingly, MCP1 secretion has been suggested to primarily be from adipocyte progenitors *in vivo*, not adipocytes themselves [44]. Therefore, while MCP1 is a known potent macrophage attractant, alterations in MCP1 (or lack thereof) likely did not lead to the decrease in adipose macrophage presence in the A-Nicastrin animals, which would have normal preadipocytes. Future work examining other cell line and cytokine changes will be needed to determine the root cause of the decrease in local macrophages, i.e. through alterations in IL-6 *trans* signalling.

Therefore, our current work is consistent adipocyte IL-6 expression, secretion, and function in adipose inflammation being regulated in part via pathways utilizing γ-secretase. Further exploration into the specific mechanisms of how adipocytes can alter the activation of ATMs, which serve a variety of roles in both the lean and obese states [9], is warranted as γ-secretase inhibition appears to profoundly affect local adipose inflammation.
